# Supplementary material for: Complexome profiling of the Chlamydomonas psb28 mutant reveals TEF5 as an early PSII assembly factor
Source: Plant Cell. 2025 Mar 17;37(6):koaf055. doi: 10.1093/plcell/koaf055 (PMC12164590; doi:10.1093/plcell/koaf055)
Supplement: koaf055_Supplementary_Data [file koaf055_supplementary_data.zip › psb28_interactive.zip!/plots/intensity/qualityPlots/peptides/RAB7_Cre06.g311900.t1.2.html]

### Cre06.g311900.t1.2

Trivial name: RAB7  
SeqCoverage [median]: 42.70  
Mass: 23.14 kDa  
  
 

| Sequence | MissedCleavage | Mass [Da] | wtIntensity | muIntensity | Score |
| --- | --- | --- | --- | --- | --- |
| ATIGADFLTK | 0 | 1035.56 | 2023.48 | 13542.58 | 86.90 |
| EIEVDDKK | 1 | 974.49 | 741.23 | 829.70 | 107.29 |
| FQSLGSAFYR | 0 | 1174.58 | 533.94 | 6236.07 | 101.54 |
| IDENGGSSR | 0 | 933.42 | 0.00 | 1166.10 | 120.87 |
| LLKVIILGDSGVGK | 1 | 1410.88 | 623.44 | 500.18 | 33.69 |
| NALRNEKEEELFMPDAVDMNTTATQR | 2 | 3022.41 | 0.00 | 2096.83 | 89.83 |
| TSLMNQYVQK | 0 | 1210.60 | 0.00 | 3436.16 | 157.91 |
| TSLMNQYVQKK | 1 | 1338.70 | 779.19 | 0.00 | 87.30 |
| VIILGDSGVGK | 0 | 1056.62 | 7826.19 | 16636.78 | 95.36 |
|  |  |  | 12527.5 | 44444.4 |  |
